# Complete reperfusion is required for maximal benefits of mechanical thrombectomy in stroke patients

**DOI:** 10.1038/s41598-017-11946-y

**Published:** 2017-09-14

**Authors:** Ángel Chamorro, Jordi Blasco, Antonio López, Sergio Amaro, Luis San Román, Laura Llull, Arturo Renú, Salvatore Rudilosso, Carlos Laredo, Victor Obach, Xabier Urra, Anna M. Planas, Enrique C. Leira, Juan Macho

**Affiliations:** 1 Comprehensive Stroke Center, Department of Neuroscience, Hospital Clinic, University of Barcelona and August Pi I Sunyer Biomedical Research Institute (IDIBAPS), Barcelona, Spain; 2 Radiology Department, Hospital Clinic, Barcelona, Spain; 3 Department of Brain Ischemia and Neurodegeneration, Institute for Biomedical Research of Barcelona, Spanish Research Council, Barcelona, Spain, IDIBAPS, Barcelona, Spain; 4 Division of Cerebrovascular Diseases, Department of Neurology, University of Iowa Carver College of Medicine, Iowa City, Iowa USA

## Abstract

A mTICI 2b or a mTICI 3 score are currently considered success following mechanical thrombectomy (MT) in acute stroke but is undetermined whether the two scores translate equivalent outcomes. We present a single-center, retrospective cohort of patients with anterior circulation stroke treated with MT and achieving a final mTICI score 2b or 3. A multimodal CT at baseline and a multimodal MRI at 24 hours assessed the growth of the infarct, and the modified Rankin Scale (mRS) assessed functional outcome at 90 days. The primary outcome was the shift analysis of the mRS at day 90 in ordinal regression adjusted for covariates (age, sex, pretreatment NIHSS score, target occlusion, infarct core, pretreatment alteplase), and the collateral score. Infarct growth was explored in a similarly adjusted multiple linear regression model. MT was started within a median of 285 minutes of symptom onset; 51 (41%) patients achieved a mTICI 2b, and 74 (59%), a mTICI 3. mTICI 3 resulted in better mRS score transitions than mTICI 2b (odds ratio 2.018 [95% CI 1.033–3.945], p = 0. 040), and reduced infarct growth (p = 0.002). We conclude that in patients with acute stroke receiving MT, success should be redefined as achieving a mTICI 3 score.

## Introduction

Mechanical thrombectomy (MT) therapy was of clinical value in selected patients with acute stroke secondary to large vessel occlusion evaluated in recent endovascular trials but the procedure yielded relevant clinical benefits in less than half of MT-treated patients^1–5^. In correspondence with these suboptimal clinical results, the endovascular trials did also show that MT resulted in complete restoration of brain reperfusion in only about one third of the patients^1–5^, highlighting the unmet need of identifying more effective strategies to improve brain reperfusion following MT.

The Modified Treatment In Cerebral Ischemia (mTICI) is recommended as the primary reperfusion scale to assess the therapeutic intervention in patients receiving MT for it was specifically designed for the cerebral circulation, has good inter-rater reliability, and strongly predicts clinical outcome^6, 7^. In this scale, mTICI score 3 defines complete reperfusion of the target downstream territory (TDT) and mTICI 2b score defines restoration of more than half of the TDT^8, 9^. Other investigators have proposed an additional TICI 2c category, defined as “near complete perfusion except for slow flow in a few distal cortical vessels, or presence of small distal cortical emboli”^10^, but the use of this category has been criticized for having a too vague definition criteria which make it prone for bias^11^.

The current guideline for healthcare professionals from the American Heart Association/American Stroke Association (AHA/ASA) recommends achieving a mTICI 2b or a mTICI 3 score following MT, on the assumption that both angiographic technical goals maximize the probability of a good functional clinical outcome^12^.

However, given the marked differences of the extent of TDT reperfused by each of these two mTICI scores, and the accepted principle of a graded association between the mTICI score and the degree of clinical benefit^13, 14^, it is necessary to determine whether the magnitude of the benefits derived from each of these angiographic scores are truly similar. Meanwhile, recent observations suggested conflicting results in MT-treated patients, for mTICI 3 score was associated^11, 15, 16^ or not^17, 18^, with a significant better outcome than a mTICI 2b score but these studies pooled data across different occlusion sites, and did not report the course of the infarction using advanced brain imaging techniques. For establishing whether mTICI 3 is or not superior to mTICI 2b in improving the functional outcome of MT-treated patients may modify current practice recommendations on which angiographic results constitute success in treating these patients, we performed the current study that incorporated the use of advanced brain imaging techniques, to compare clinical outcomes with the evolution of the brain infarction.

## Methods

### Subjects

Single-center retrospective cohort of prospectively collected patients treated with MT between the years 2010–2016 at the Hospital Clinic of Barcelona, a tertiary academic center serving a population of 2.2 M inhabitants. Inclusion criteria included (1) Anterior circulation strokes, with arterial occlusions located at the terminal intracranial internal carotid artery (ICA), M1-M2 segments of the middle cerebral artery (MCA), or tandem lesions; (2) Patient underwent multimodal brain CT scan assessment, before MT, multimodal brain MRI assessment, at 24 h after symptom onset, and functional assessment using the modified Rankin scale (mRS), at day 90; and (3) Patients achieved complete local recanalization of the arterial occlusion and a distal reperfusion mTICI score of 2b or 3 at the end of the procedure^6, 7^. The qualifying strokes were classified according to the Trial of Org 10172 in Acute Stroke Treatment (TOAST) criteria after a complete diagnostic workup^19^. IV alteplase was given within 4.5 hours from stroke onset following current recommendations and the treatment was interrupted at the time of the initiation of the endovascular procedure to minimize the risk of bleeding complications. All thrombectomy devices used in these patients were stent-retrievers, local intra-arterial infusion of alteplase was not used and the procedures were performed without sedation. If possible, distal lesions were attempted first in patients with tandem lesions. Stents were placed after therapy of cervical lesions and the antiplatelet regime included 600 mg IV ASA plus 300 mg oral clopidogrel, at the start of the mechanical procedure, followed by a daily oral dose of 100 mg ASA + 75 mg clopidogrel, for three months. After MT, patients were transferred to the Stroke Unit and were managed following European Stroke Organization (ESO) Guidelines. Data collected included demographics, risk factors, National Institute of Health Stroke Scale (NIHSS) scores during hospitalization, mRS scores, angiography, CT and MRI variables. We also recorded the total number of thrombectomy device passes, and workflow metrics that included the time delay from symptom onset or from last time seen well to the end of the endovascular procedure. The presence of early ischemic changes was assessed using the Alberta Stroke Program Early CT Score (ASPECTS). Participants in the study consented for storage of their data in a local database for the purpose of research that was declared into a Web-based registry that satisfied all legal requirements for protection of personal data, for monitoring by the Catalan Health Department^20^. The Local Ethics Committee approved the research that was conducted according to the principles of the Declaration of Helsinki. Physicians not involved in the acute phase management of the patients and blinded to the final mTICI score assessed the functional outcome with the use of the mRS and following a structured questionnaire, at 90 days. “Excellent” outcome was defined as a mRS score 0 to 1, at day 90. “Early dramatic recovery“ was defined as a reduction of at least 8 points in the NIHSS at 24 hours from the baseline NIHSS score, or a NIHSS score ≤2 at 24 hours of stroke onset. Bleeding complications were scored on CT or MRI according to the European Cooperative Acute Stroke Study (ECASS) criteria^21^, and symptomatic intracerebral hemorrhage defined the presence of blood on brain imaging associated with an increment of at least 4 points in the NIHSS score.

### Brain imaging assessment

The imaging protocol integrated a multimodal whole-brain CT scan, including a non-contrast CT (NCCT), CT angiography (CTA) and CT perfusion (CTP), which were performed at hospital admission using a SIEMENS Somatom Definition Flash unit, as reported^22^. Pre-treatment perfusion evaluation was performed using a commercially available semi-automated perfusion analysis software (Siemens) based on the maximum slope model of perfusion. The infarct core was segmented based on a cerebral blood volume (CBV) threshold of 0.6 relative to the contralateral white matter and defined as “malignant” if this core was higher than 70 ml. The ischemic penumbra was segmented based on a Time to Peak (TTP) relative threshold of 6 seconds compared to contralateral hemisphere for identification of critically hypoperfused tissue. The collateral circulation was scored on CTA according to a validated grading system that ranged from 0 to 3^23^.

At 24 hours of stroke onset, a multimodal 1.5 T brain MRI protocol was performed and included a diffusion-weighted image (DWI) sequence obtained with RT = 12600ms, ET = 89ms, matrix of 192 × 192, FoV = 230mm, voxel size = 1.2 × .2 × 3 mm3 (x, y and z directions), and b-values = 0 and 1000 mm/s2. DWI lesion volumes were calculated using AMIRA software by means of a semi-automated thresholding method to identify regions of interests with high DWI signal intensity (exceeding the intensity of the contralateral hemisphere by more than three standard deviations). Infarct growth was defined on brain imaging as the difference between 24-hour DWI infarct volume and baseline nonviable tissue volume on CTP. For infarct growth measurements, CTP maps were recalculated by commercial software MIStar (Apollo Medical Imaging Technology, Melbourne, Australia) using singular value decomposition with a delay correction and nonviable tissue was measured using a relative threshold of 30% of the mean Cerebral Blood Flow value in the unaffected/contralateral hemisphere.

We used the mTICI score to assess antegrade brain perfusion past the occlusion into the distal arterial bed and terminal branches. The mTICI scale distinguishes no perfusion (mTICI grade 0), minimal flow past the occlusion but no perfusion (mTICI grade 1), partial reperfusion of less than half of the TDT (mTICI grade 2a), partial reperfusion of more than half of the TDT (mTICI grade 2b), and complete reperfusion without any flow defects^6, 8, 9^. Experienced radiologists and interventionalists blinded to all clinical data assessed the brain imaging data in the study.

### Statistical analysis

Continuous variables were reported as mean with standard deviations (SD) or median with interquartile ranges (IQR) and were compared with the Student t-test, or Mann–Whitney U test as appropriate. Categorical variables were compared with the Chi-square and Fisher exact tests. The primary outcome of the study was the shift analysis of the mRS at day 90 according to the final mTICI score and it was analyzed using ordinal regression and grouping categories 5 (severe disability) and 6 (death). Logistic regression models were used to evaluate the independent predictors of the final mTICI score and its independent effect on the secondary outcomes of the study, including the proportions of patients with excellent outcome at 90 days and the rate of early dramatic recovery at 24 hours. In the logistic and ordinal models, dependent covariates were selected as per the HERMES Collaboration^24^, including age (a continuous variable), sex (binary variable), pretreatment NIHSS score (discrete variable), target occlusion location (a 4-level categorical variable—ICA, M1, M2, Tandem), ASPECTS (discrete variable), and pretreatment intravenous alteplase (binary variable). We further included in the multivariate models the independent effect of the collateral score^23^ (binary variable - scores 0 to 1 were classified as “poor collaterals” and score 2 to 3 as “good collaterals”). The independent effect of the final mTICI score on the volume of the infarct at 24 h and the rate of infarct growth on multimodal imaging at 24 hours was explored in multiple linear regression models adjusted for the aforementioned covariates. The analysis was performed using SPSS Version 19.0, and the level of significance was established at a 0.05 level (2-sided).

### Data availability

The data reported in this manuscript is available for an external audit.

## Results

Between March 2010 and May 2016, 125 of 347 (36%) patients treated with MT at our institution met the entry criteria of the study. Contrarily, 222 patients were excluded for (1) a posterior circulation stroke (n = 31); (2) lost to follow-up due to transfer to a referral Primary Stroke Center after MT (n = 113); (3) unavailability of multimodal brain imaging (n = 37); or (4) mTICI 2a/1/0 score at the end of MT (n = 41). The comparability of the excluded patients with the final study population is shown in Supplemental Table 1.

### Baseline characteristics of the study population

Recanalization of the local occlusion occurred within a median (IQR) of 285 (210–369) minutes of symptom onset; 51 (41%) patients achieved a mTICI 2b score and 74 (59%) patients a mTICI 3 score. Patients with final mTICI 2b or 3 scores did not show significant differences in demographics, risk factors, target occlusion location, use of bridging intravenous alteplase before MT, or size of infarct core calculated either with the ASPECTS on NCCT or on CTP (Table 1). Expectedly, a mTICI 3 score was associated with shorter time to recanalization from stroke onset, and less number of device passes (Table 1). A final mTICI score 3 was more frequent in patients with good leptomeningeal collateral scores (Table 1), and this association was highly significant in a multivariate model adjusted for the predefined covariates of the study, (odds ratio 2.765 [95% CI 1.248–6.123]; p = 0. 012).

**Table 1:** General traits of the study population.

	mTICI 3	mTICI 2b	P value
N (%)	74 (59)	51 (41)	—
Baseline traits			
Age yr, median	71 (63–79)	69 (59–81)	0.956
Males, n (%)	38 (51)	28 (55)	0.696
Hypertension, n (%)	37 (50)	27 (53)	0.746
Diabetes, n (%)	12 (16)	8 (16)	0.937
Dyslipemia, n (%)	28 (38)	19 (37)	0.947
CAD, n (%)	11 (15)	8 (16)	0.703
Atrial fibrillation, n (%)	19 (26)	16 (31)	0.486
Smoking, n (%)	15 (20)	8 (16)	0.516
Stroke subtype, n (%)			0.901
Large vessel disease	20 (27)	13 (25)	
Cardioembolism	27 (37)	18 (35)	
Undetermined etiology	18 (24)	11 (22)	
Other	9 (12)	9 (18)	
Pretreatment NIHSS, median (IQR)	15 (11–19)	17 (14–21)	0.081
Workflows and radiological findings			
Vessel occlusion on CTA, n (%)			0.566
Tandem	14 (19)	7 (14)	
ICA	9 (12)	10 (20)	
M1	39 (53)	28 (55)	
M2	12 (16)	6 (12)	
Time to CTP, min, median	170 (90–215)	196 (93–286)	0.207
Time to groin puncture, min, median	233 (164–295)	270 (186–365)	0.047
Time to recanalization, min, median (IQR)	267 (201–3338)	332 (229–422)	0.012
Number of passes	2 (1–3)	2 (1–4)	0.004
Previous use of intravenous alteplase, n (%)	46 (62)	33 (65)	0.772
Collateral score, n (%)			0.010
0–1 (poor)	16 (22)	22 (43)	
2–3 (good)	58 (78)	29 (57)	
Baseline CTP infarct core, ml, median	16.6 (7.4–32,9)	24.3 (9.3–38.1)	0.236
NCCT ASPECTS, n (%)			0.156
0–6	0	2 (4)	
7–8	23 (31)	19 (37)	
9–10	51 (69)	30 (59)	

TICI = Thrombolysis In Cerebral Infarction; DWI = Diffusion weighted imaging; mRS = modified Rankin scale; ASPECTS = Alberta Stroke Program Early CT score; NCCT = Non contrast CT scan; CTP = CT Perfusion.

### A final mTICI 3 score was associated with improved clinical outcomes

**Figure 1:**
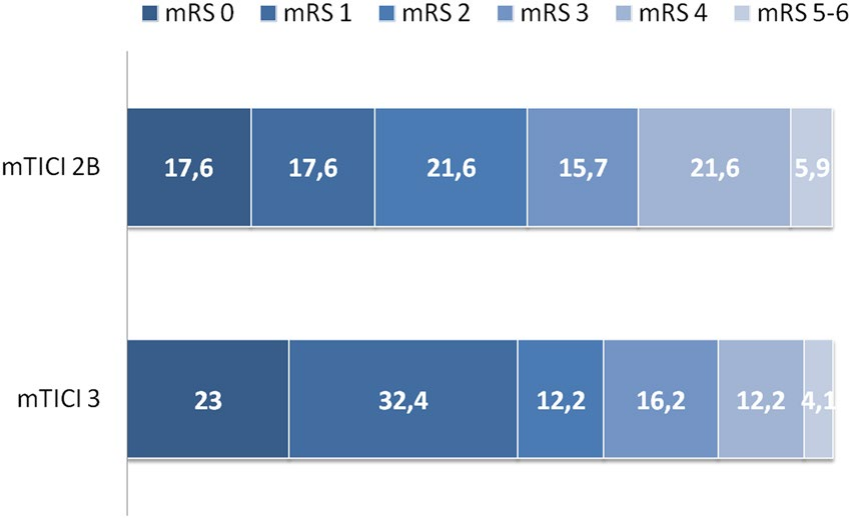
Distribution of mRS scores at 90 days. Proportions of patients within each score category on the 7-point scale (where 0 indicates no symptoms and 6 indicates death) at 90 days, by mTICI score. mRS = modified Rankin Scale.

The primary outcome measure of the study showed that more patients with mTICI 3 were in a better score category on the mRS at 90 days than were patients with mTICI 2b, and this difference was statistically significant in ordinal regression analysis adjusted for confounders (odds ratio 2.018 [95% CI 1.033–3.945]; p = 0. 040, Fig. 1). Age, and pretreatment NIHSS score, but not the remainder covariates, were also significant independent outcome predictors.

Excellent outcome at 90 days was reported in 18 (35%) of 51 patients achieving a mTICI 2b score and in 41 (55%) of 74 patients achieving a mTICI 3 score, (adjusted odds ratio 2.739 [95% CI 1.124–6.182]; p = 0.015). Early dramatic recovery at 24 hours, was diagnosed in 25 (49%) patients with mTICI 2b and in 54 (73%) patients with mTICI 3, (adjusted odds ratio 3.078 [95% CI 1.384–6.849]; p = 0.006). Finally, the mortality and the rate of symptomatic intracerebral hemorrhage did not differ between patients with mTICI 3 or 2b scores (Table 2).

**Table 2:** Clinical end-points according to the final mTICI score.

	mTICI 3	mTICI 2b	P Value
N	74	51	—
mRS 0 to 1 day 90, n (%)	41 (55)	18 (35)	0.027
Early dramatic recovery^a^, n (%)	54 (73)	25 (49)	0.006
Mortality, n (%)	3 (4.1)	2 (3.9)	0.970
Symptomatic intracerebral hemorrhage, n (%)	0	2 (3.9)	0.086

^a^≤8points or NIHSS score < 2 at 24 hours; mTICI = modified Thrombolysis In Cerebral Infarction; mRS = modified Rankin scale.

### A final mTICI 3 was associated with improved surrogate brain imaging findings

**Figure 2:**
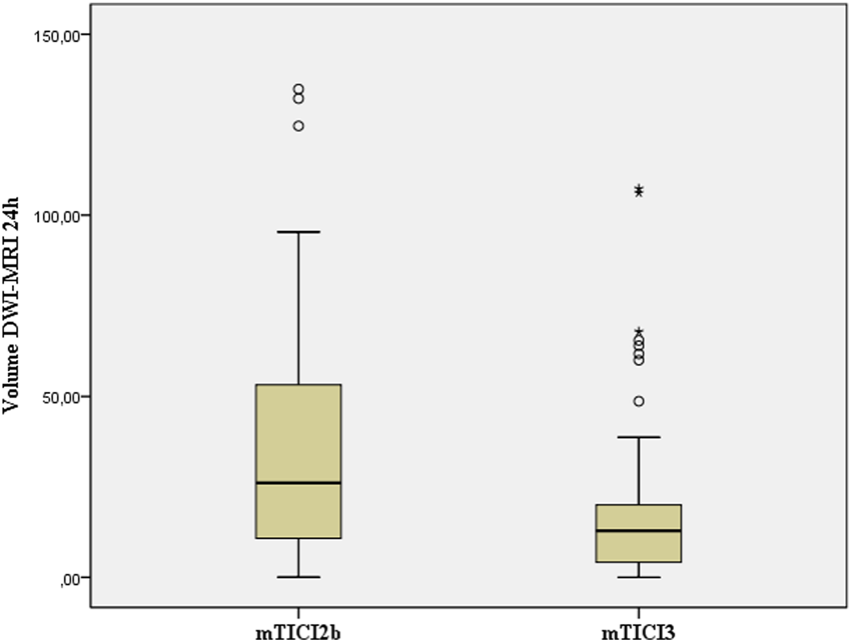
Box-whisker plot of infarct volume on DWI-MRI at 24 hours of stroke onset in 51 patients with mTICI 2b and 74 with mTICI 3 (boxes, 25 to 75% interquartile range [IQR]; central horizontal bars, median; outer horizontal bars, 10 to 90% IQR).

Median (IQR) infarct volume on DWI MRI at 24 hours of stroke onset was smaller in patients with mTICI 3 score compared with patients with mTICI 2b score (12.84 [4.15 to 20.00] ml versus 26.11 [10.66 to 55.06] ml, Mann Whitney U test P < 0.001) (Fig. 2). Correspondingly, median (IQR) infarct growth at 24 hours was also reduced in patients with mTICI 3 score compared with patients with mTICI 2b score (−2.62 [−16.46 to 5.59] ml versus 2.29 [−18.64 to 31.56] ml, Mann Whitney U test P = 0.060). In linear regression models adjusted for the predefined covariates of the study, the rate of infarct growth at 24 hours was significantly reduced in patients with a final mTICI 3 score (regression coefficient −25,5 [95% CI −41.8 to −9.1]; p = 0.003) ml. The results of these regression models did not change by adjusting for the ASPECTS on NCCT, the core of infarct on CTP or the time to recanalization (data not shown).

## Discussion

This study demonstrated that obtaining a final mTICI 3 score at the end of MT doubled the odds of improving functional outcomes over the entire range of health transition across the categories of the mRS compared with a mTICI 2b score. Although there were some basal differences between patients that developed a TICI3 or TICI2b score at the end of the procedure, the superior benefit of TICI 3 continued after accounting for the effect of age, sex, baseline severity of stroke, target occlusion, baseline infarction core, use of bridging intravenous alteplase and collateral status. Consistently, a mTICI 3 score was also superior to a mTICI 2b score to increase the rate of excellent outcome at 90 days, and the proportion of patients with early dramatic recovery at 24 hours, while there were no differences in mortality or in the rate of symptomatic intracerebral hemorrhage. Altogether, these results underscore the relevance of achieving a full reperfusion after MT and dispute current practice recommendations of considering mTICI 2b and 3 scores equally acceptable technical results following MT^12^. In patients with acute stroke receiving MT, success should be redefined as achieving mTICI 3, and the treating physicians should not be satisfied with lesser degrees of reperfusion. Nonetheless, the goal of endovascular treatment should not be only the mTICI score, but also the implementation of faster and more efficient devices in order to obtain better recanalization rates.

This study also evaluated longitudinally the volume of the infarction core using advanced brain imaging techniques that allowed a reliable distinction between reversible ischemia and brain infarction^25^. Our finding that a mTICI 3 score was more effective than a mTICI 2b score to limit infarct growth and infarct volume, is consistent with a more efficient salvage of the ischemic penumbra. The marked increment in the rate of early dramatic clinical recovery amongst patients with a mTICI 3 lend additional clinical support to the salvage of the penumbra as the main driver of its additional benefits compared with the mTICI 2b score.

Expectedly, mTICI 3 procedure times were shorter with fewer passes than mTICI 2b but we also found that mTICI 3 was more likely in patients with better baseline collateral status. The latter finding extended previous observations correlating reperfusion success with the collateral score^26^, but also showed that attaining complete reperfusion after MT maximized the clinical benefits, irrespective of the collateral score or the baseline infarct core on brain imaging.

In experimental brain ischemia, incomplete distal brain reperfusion mainly result from capillary or perivascular clogging, distal microembolism and oxidative stress^27–29^. To what extent each of these mechanisms limited brain reperfusion in this study remains undetermined. Nonetheless, the greatest benefits encountered in the study in relation to a mTICI 3 score support exploring new therapeutic approaches in patients with a mTICI 2b score at the end of MT. Considering the possible biological nature of incomplete brain reperfusion^27^, sensible strategies would include rescue intraarterial thrombolysis^12^, and uric acid therapy^30^.

The study also has limitations that deserve comment. It was conducted in a single comprehensive stroke center that was mandated by local health policies to return one third of the treated patients to local community hospitals after MT, which impeded follow-up of these patients. However, such transfer was solely based on place of residence and the analysis of the excluded patients did not reveal significant baseline differences with the study population, which lessens the concern for selection bias. While single center studies may limit their external validity, a single-center approach may be a strength to minimize the noise derived from ancillary care practice and procedural variability.

## Conclusions

This study demonstrated the relevance of achieving a mTICI 3 score at the end of MT to maximize the functional benefits of brain reperfusion. Compared with patients with a mTICI 2b score, patients who achieved a mTICI 3 had better overall health transitions in the full range of the mRS, increased proportions of excellent outcome and early dramatic recovery, less infarct growth and smaller final infarcts. Altogether, these results justify the search of more effective reperfusion therapies and call for a change of current practice recommendations in patients treated with MT indicating that only a mTICI 3 angiographic score should be considered success after MT.

## Electronic supplementary material

Supplementary  Information
