# Supplementary material for: Complete reperfusion is required for maximal benefits of mechanical thrombectomy in stroke patients
Source: Sci Rep. 2017 Sep 14;7:11636. doi: 10.1038/s41598-017-11946-y (PMC5599658; doi:10.1038/s41598-017-11946-y)
Supplement: Supplementary file 1 — Supplementary Information [file 41598_2017_11946_MOESM1_ESM.pdf]

Supplementary material

Title

Complete reperfusion is required for maximal benefits of mechanical thrombectomy in stroke patients

Authors

Ángel Chamorro, MD, PhD[1]; Jordi Blasco, MD[2]; Antonio López, MD[2]; Sergio Amaro MD, PhD [1]; Luis San Román, MD, PhD [2]; Laura Llull MD, PhD [1]; Arturo Renú MD[1]; Salvatore Rudilosso, MD[1]; Carlos Laredo PhD.[1]; Victor Obach, MD.[1]; Xabier Urra, MD, PhD [1];Anna M. Planas PhD.[3]; Enrique C. Leira, MD, MS [4];Juan Macho,MD,PhD [2]

Supplemental Table I Main baseline traits of the patients excluded from the study

	**Patients included**	**Patients excluded**	**P value**
N	125	222	-
Baseline traits			
Age yr, median	70 (61-80)	72 (63-79)	0.995
Males, %	53	48	0.366
Hypertension,%	51	57	0.290
Diabetes, %	16	25	0.050
Dyslipemia,%	38	39	0.815
CAD, %	15	19	0.341
Atrial fibrillation, %	28	34	0.304
Smoking, %	18	22	0.422
Pretreatment NIHSS, median (IQR)	16 (13-20)	17 (13-21)	0.237
NCCT ASPECTS >6, , median (IQR)	9 (8-10)	9 (8-10)	0.153
ASPECTS=Alberta Stroke Program Early CT score; NCCT = Non contrast CT scan			
